# Structure Elucidation and Biological Evaluation of Maitotoxin-3, a Homologue of Gambierone, from *Gambierdiscus belizeanus*

**DOI:** 10.3390/toxins11020079

**Published:** 2019-02-01

**Authors:** Andrea Boente-Juncal, Mercedes Álvarez, Álvaro Antelo, Inés Rodríguez, Kevin Calabro, Carmen Vale, Olivier P. Thomas, Luis M. Botana

**Affiliations:** 1 Departamento de Farmacología, Farmacia y Tecnología Farmacéutica, Facultad de Veterinaria, Universidad de Santiago de Compostela, 27002 Lugo, Spain; andrea.boente.juncal@usc.es (A.B.-J.); mdelcarmen.vale@usc.es (C.V.); 2 Laboratorio CIFGA S.A., Plaza de Santo Domingo no. 20, 5a planta, 27001 Lugo, Spain; mercedes@cifga.es (M.Á.); alvaro@cifga.es (Á.A.); inesrguez@cifga.es (I.R.); 3 Marine Biodiscovery, School of Chemistry and Ryan Institute, National University of Ireland Galway (NUI Galway), University Road, H91 TK33 Galway, Ireland; kevin.calabro@nuigalway.ie (K.C.); olivier.thomas@nuigalway.ie (O.P.T.)

**Keywords:** maitotoxin-3, 44-methylgambierone, gambierone, maitotoxin, *Gambierdiscus belizeanus*, cytosolic calcium concentration, glutamate receptors, neurotoxicity, ciguatera

## Abstract

*Gambierdiscus* species are the producers of the marine toxins ciguatoxins and maitotoxins which cause worldwide human intoxications recognized as Ciguatera Fish Poisoning. A deep chemical investigation of a cultured strain of *G. belizeanus*, collected in the Caribbean Sea, led to the identification of a structural homologue of the recently described gambierone isolated from the same strain. The structure was elucidated mainly by comparison of NMR and MS data with those of gambierone and ascertained by 2D NMR data analyses. Gratifyingly, a close inspection of the MS data of the new 44-methylgambierone suggests that this toxin would actually correspond to the structure of maitotoxin-3 (MTX3, *m*/*z* 1039.4957 for the protonated adduct) detected in 1994 in a Pacific strain of *Gambierdiscus* and recently shown in routine monitoring programs. Therefore, this work provides for the first time the chemical identification of the MTX3 molecule by NMR. Furthermore, biological data confirmed the similar activities of both gambierone and 44-methylgambierone. Both gambierone and MTX3 induced a small increase in the cytosolic calcium concentration but only MTX3 caused cell cytotoxicity at micromolar concentrations. Moreover, chronic exposure of human cortical neurons to either gambierone or MTX3 altered the expression of ionotropic glutamate receptors, an effect already described before for the synthetic ciguatoxin CTX3C. However, even when gambierone and MTX3 affected glutamate receptor expression in a similar manner their effect on receptor expression differed from that of CTX3C, since both toxins decreased AMPA receptor levels while increasing N-methyl-d-aspartate (NMDA) receptor protein. Thus, further studies should be pursued to clarify the similarities and differences in the biological activity between the known ciguatoxins and the new identified molecule as well as its contribution to the neurological symptoms of ciguatera.

## 1. Introduction

Ciguatera Fish Poisoning (CFP) is a human foodborne illness caused by the ingestion of marine fish contaminated with ciguatoxins (CTX) and other structurally related ladder-shaped polyether toxins traditionally identified as maitotoxins (MTX) [1]. Ciguatera has recently become a threat to fish consumers in non-endemic regions mainly due to the expanding international trade in seafood from tropical fisheries and to the proliferation of *Gambierdiscus* as a consequence of ocean warming and climate change [2,3,4]. Human intoxications by ciguatera affect annually 10,000 to 500,000 people worldwide [4], although the prevalence of this disease could be higher [3,4]. Traditionally, CFP was thought to affect mainly tropical and sub-tropical areas but presently it has expanded worldwide [2]. In fact, the presence of *Gambierdiscus* was previously associated with tropical areas, but presently CFP intoxications have been identified in Europe during recent decades, especially in the Canary Islands [5,6] and Madeira [7,8].

Maitotoxins are among the largest natural non-polymeric compounds and the most toxic marine compounds identified to date [9]. These toxins are produced by dinoflagellates of the genera *Gambierdiscus* and *Fukuyoa* [10,11]. Up to now, four toxin analogs designated as maitotoxin-1 (MTX1), maitotoxin-2 (MTX2), maitotoxin-3 (MTX3) and maitotoxin-4 (MTX4) isolated from different strains of these dinoflagellates have been identified in this toxin group [1,10,12,13]. So far, maitotoxin (MTX1) is the largest non-polymeric carbon chain molecule in nature [14,15]. Additionally, species of *Gambierdiscus* and *Fukuyoa* have been shown to produce other non-structurally related polyether analogs such as gambieric acids [16], gambieroxide [17], gambierol [18], and the recently identified gambierone [19]. The diversity in the chemical structures and biological activities of the molecules involved in CFP could reflect their different mechanisms of action. Thus, while MTX-like activity is associated with a massive calcium influx and rapid cell death [20,21,22,23], ciguatoxins and gambierone cause voltage-gated sodium channel activation at negative membrane potentials leading to cell depolarization at rest and to the disruption of peripheral and central nerve transmission [3,19]. Finally, gambierol acts mainly by blocking voltage dependent potassium channels [24,25].

Among the toxins involved in CFP in Australia, a putative maitotoxin-3 (p-MTX3) was frequently encountered in most species of *Gambierdiscus* [26]. For many years, p-MTX3 has been detected in *Gambierdiscus* species that showed low toxicities in functional bioassays [27,28], but the chemical structure of this molecule remained to be elucidated due to the low amount of biomass available. Mass spectrometry analyses of strains of *Gambierdiscus* revealed the presence of p-MTX3 with a major MS peak at *m/z* 1039.5 [29]. Presently, routine monitoring of toxins (particularly ciguatoxins) by mass spectrometry revealed the almost ubiquitous presence of this mass in positive ionization mode [27,28,30]. MTX3 was initially isolated by Holmes et al. [10] from cultures of the *Gambierdiscus* strain WC1/1. The molecule was depicted as di-sulfated with a MW = 1060.5 Da (for di-sodium salt) and *m/z* 1039.5 as the most intense peak observed in IonSpray Mass Spectrometry (ISMS). MTX3 was found to be toxic to mice [10] and despite their considerable differences in molecular size (maitotoxin-3 is about one third the MW of maitotoxin-1), both molecules showed similar in vivo activity in mice for their death-time vs. intraperitoneal dose [10]. Recently, the structure of a new maitotoxin analog, named MTX4, was identified from a strain of *Gambierdiscus excentricus*. MTX4 was reported to exhibit a toxic effect similar to the one of MTX1 in neuroblastoma cells [13]. The presence of the p-MTX3 with the same MS data was also described in this strain but again no chemical structure could be assigned. Initial studies indicated that MTX3 produced similar symptoms and biological effects to those elicited by MTX1 and MTX2 although with lower potency than MTX1 [10]. The reduction in potency after solvolysis of these maitotoxins led to suggests that sulfate moieties were important for the biological activity of each maitotoxin analog [10,29].

So far, five compounds produced by *Gambierdiscus* were found to contain at least one sulfate group: MTX1, MTX2, MTX3, gambierone and gambieroxide. The presence of a sulfate group in the molecular skeleton of maitotoxin is not synonymous of a typical maitotoxin-like biological activity. In fact, recent analysis of the biological activity of gambierone revealed that this compound did not exhibit similar cellular effects to those of maitotoxin. Rather, the biological activity of gambierone resembled the effect of the voltage-gated sodium channel activator CTX3C although the biological potency of gambierone was lower than that of the synthetic ciguatoxin [19]. The biological activity of gambieroxide, the other sulfate-containing polyether compound related to maitotoxin, has not yet been assessed.

**Figure 1:**
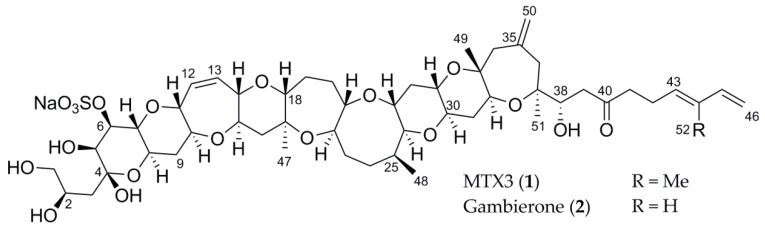
Structure of the metabolites isolated from *Gambierdiscus belizeanus*.

During our efforts to isolate ciguatoxin from *G. belizeanus* we found a large chemical diversity and we decided to focus on two major metabolites with two prominent ions at *m/z* 1025.6 and 1039.5 in electrospray ionization (ESI)(+)-MS. The metabolite with the first mass has recently been identified as a novel ladder-shaped polyether compound named gambierone [19]. The other ion was eluted in the maitotoxin region in a typical reverse phase chromatography. We report herein the structure of the metabolite associated with the mass at *m/z* 1039.5. All NMR data are consistent with 44-methylgambierone (1) (Figure 1). This structure therefore represents the candidate structure for MTX3. We also report the comparative biological activities of this new metabolite and gambierone both isolated from *G. belizeanus*.

## 2. Results

*Gambierdiscus belizeanus* was grown in 20 L bags and scaled up to 2000 L. The cells were harvested by filtration and extracted with MeOH under ultrasonication. The methanolic extract was then subjected to successive separation steps and ultimately purified by HPLC using a Reverse Phase (RP) phenylhexyl column yielding pure compound **1** (6 mg) and gambierone (**2**, 15 mg).

### 2.1. Structure Elucidation of Compound 1

Compound **1** was isolated as a white solid with the molecular formula C_52_H_78_O_19_S as deduced from High Resolution Mass Spectra (HRMS) data with a major ion peak at *m*/*z* 1039.4957 [M+H]^+^. A first inspection of the ^1^H NMR spectrum of **1** in CD_3_OD confirmed its polyketide nature with the presence of oxymethine and methyl signals (Figure S1). The ^13^C and HSQC NMR spectra of **1** confirmed 52 carbon signals in the form of five methyls (four singlets and one doublet), 17 methylenes (including 14 non-oxygenated, one oxygenated, and two unsaturated), 23 methines (one non-oxygenated, 18 oxygenated and four unsaturated), one ketal at *δ*_C_ 100.7 (C-4), one ketone at *δ*_C_ 211.6 (C-40), three non-protonated and oxygenated carbons and one unsaturated quaternary carbon at *δ*_C_ 136.0 (C-44) (Table 1, Figures S2, S3 and S6). The presence of a sulfate was demonstrated by the presence of an intense fragment at *m*/*z* 941.5299 corresponding to [M+H-H_2_SO_4_]^+^ The molecular formula proposed for **1** corresponded to a structural homologue of gambierone (**2**) and indeed the fragmentation pattern was very similar between both compounds suggesting the presence of an additional methylene in the structure of **2** compared with the one of **1**. While the signals of the protons of the ladder-shaped western part of **2** were clearly maintained in **1**, some differences were observed in the eastern part of the molecule. Indeed, the lack of one olefinic proton and the presence of a new methyl at *δ*_H_ 1.74 (s, 3H, H-52) and the unsaturated quaternary carbon were shown in the HSQC NMR spectrum (Figure S6). The position of the new methyl on the olefinic carbon C-44 was established through the key H-52/C-42, C-43 and C-44 HMBC correlations (Figure S7). The relative configuration of the C-43/C-44 double bond was deduced as *E* from a key H_2_-42/H_3_-52 rOe.

**Figure 2:**
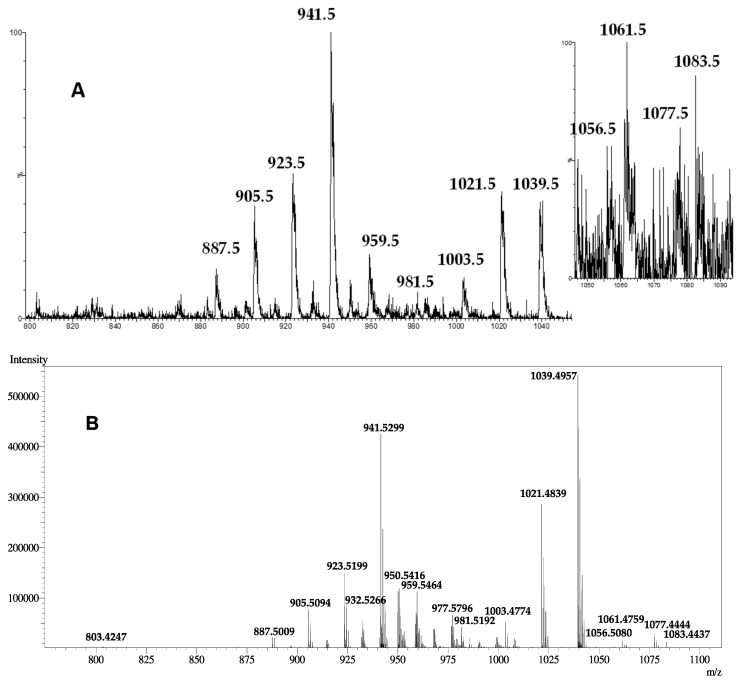
(**A**) ESI(+)-LRMS and (**B**) ESI(+)-HRMS spectra of compound **1**.

Interestingly, compound **1** shared the same major ion peak at 1039.5 in ESI(+)-LRMS with the one characterizing the putative MTX3, since 1994 and recently found in other strains of *Gambierdiscus* [31]. Therefore, we wondered whether 44-methylgambierone (**1**) could be the actual structure of the long-sought p-MTX3. First, MTX3 was always assumed to be disulfated from ESI(+)-LRMS data when **1** has been proven unambiguously to contain only one sulfate group. The molecular formula was never obtained for MTX3 as only low resolution mass spectra were given with associated fragments and therefore the presence of two sulfates was only a vague assumption [29]. The fragmentation pattern obtained in our ESI(+)-LRMS analysis of **1** is in perfect agreement with the one obtained at 120V for MTX3 in the first report of this molecule and we could actually correct the proposed ions for MTX3 (Figure 2, Table 2) [29]. We are therefore very confident to propose the structure of p-MTX3 as (43*E*)-44-methylgambierone (**1**). The absolute configuration of **1** is supposed to be the same as the one described for gambierone (**2**) based on ECD calculations due to structure similarity [19]. 

Based on MS data and their fragmentation patterns, we also propose a possible monitoring analysis for both the presence of gambierone (**2**) and MTX3 (**1**) using the following MRM transitions (Table 3).

### 2.2. Biological Activity of MTX3 and Gambierone

**Figure 3:**
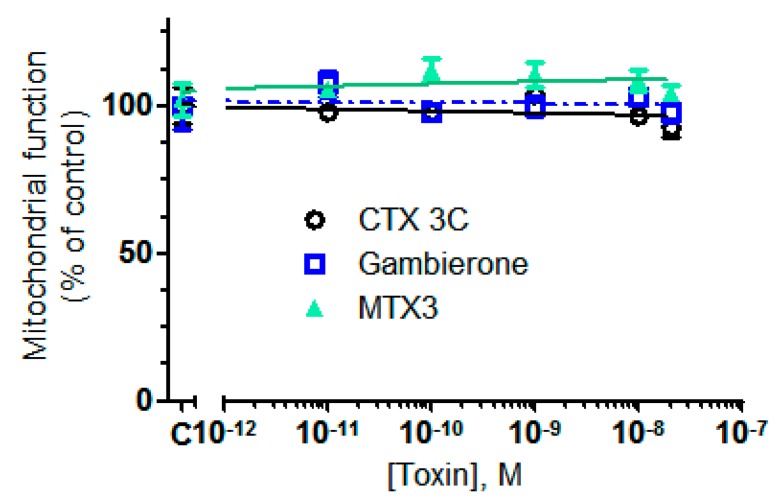
Comparison of the effects of CTX3C, gambierone and MTX3 on cell viability in human cortical neurons. None of the toxins affected CTX0E16 cell viability at low concentrations. In this case, the maximum toxin concentration evaluated was 20 nM, and toxicity was evaluated after exposure of the cells to the different compound concentrations for 5 days in vitro. Cell viability was evaluated by the MTT assay. Results are expressed as mean ± sem of 4 independent experiments, each performed in triplicate.

The biological activity of gambierone (**2**) was previously reported in undifferentiated human neuroblastoma cells and in cell lines expressing voltage dependent sodium channels isoforms [19]. In these biological systems **2** exhibited a moderate activity similar to the one observed for the synthetic ciguatoxin CTX3C although with lower potency. In this work, the biological activities of synthetic ciguatoxin CTX3C, MTX1, gambierone (**2**) and MTX3 (**1**) were compared in human neuronal cortical neurons previously used to evaluate the effects of other marine toxins in neuronal function [32,33] and in the human neuroblastoma cells used earlier [19]. As indicated in Figure 3, in vitro exposure of human cortical neurons for 5 days to **1**, **2** or CTX3C at concentrations ranging from 0.01 nM to 20 nM did not affect cellular viability in cultures of human cortical neurons. This result agrees with previous observations in primary cultures of mice cortical neurons showing no cytotoxic effect of the synthetic ciguatoxin CTX3C [22]. Furthermore, it supports the previously reported similarities between the biological activities of CTX3C and **2 [19,22]**, and additionally it reveals for the first time a similar activity for MTX3 and gambierone. It is worth highlighting the lack of cytotoxicity of these three compounds in human cortical neurons while MTX1 induced complete cell death after 2 h, 24 h or 5 days treatment with IC_50_ values below 1 nM [32]. 

**Figure 4:**
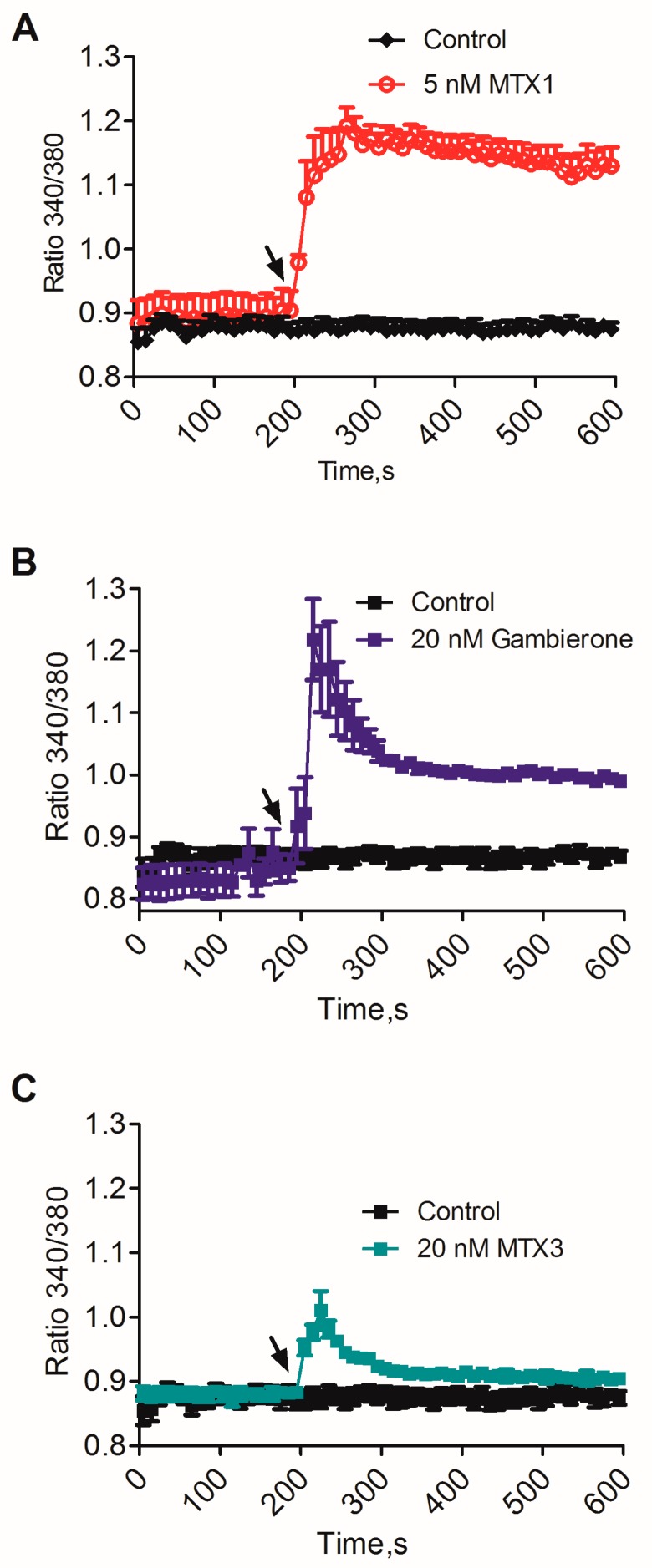
Effect of MTX1 (**A**), gambierone (**B**) and MTX3 (**C**) on the cytosolic calcium concentration in human differentiated cortical neurons. A concentration of 5 nM MTX1 caused at rapid and sustained increase in [Ca^2+^]_c_ while both gambierone and MTX3, at 20 nM, elicited a shorter and smaller effect on [Ca^2+^]_c_. Data represent means ± sem of 4 independent experiments in the case of gambierone and 3 independent experiments for MTX1 and MTX3. Bath application of the toxins is indicated by the arrow.

To complete the comparison of the biological activity of gambierone and MTX3 in the same cellular system, the effect of these compounds on the cytosolic calcium concentration ([Ca^2+^]_c_) was evaluated. Calcium homeostasis plays a main pathophysiological role in synaptic signaling and neurodegeneration [34] and a known effect of MTX1 in mice and human cortical neurons is to induce a massive calcium influx that leads to rapid neuronal death [22,33]. Therefore, the effect of gambierone and MTX3 on [Ca^2+^]_c_ was evaluated. As indicated in Figure 4, exposure of differentiated human cortical neurons to either gambierone or MTX3 at 20 nM caused a small rise in the cytosolic calcium concentration. In contrast with the fast and sustained [Ca^2+^]_c_ rise elicited by 5 nM MTX1 (Figure 4A), the effect of either gambierone (Figure 4B) and MTX3 (Figure 4C) was considerably shorter and smaller , thus confirming that the biological activity of MTX3 was of lower potency than that of MTX1 as previously demonstrated in vitro [10] although further investigations should be pursued to identify the role of this molecule on the neurological symptoms of human CFP [10].

**Figure 5:**
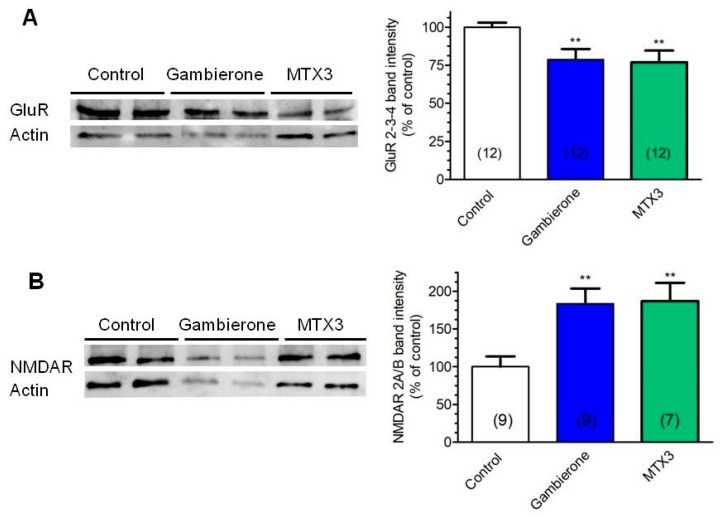
Long term exposure (15 days in culture) of human differentiated cortical neurons to either gambierone or MTX3, at 20 nM, affected the protein level of glutamate receptor subunits. (**A**) Both compounds decreased the protein level of the AMPA receptor subunits. Western blot bands showing the expression of GluR2,3,4 subunit proteins are shown on the left panel and the corresponding quantifications of the bands are shown on the right. (**B**) The same treatments were performed to evaluate the effect of the compounds on the expression of NMDA receptor subunits NR2A/B. Representative western blot bands showing NR2A/B levels in differentiated CTX0E16 neurons are shown on the left panel and band intensities quantifications are shown on the right. Fifteen days exposure of human cortical neurons to 20 nM gambierone or 20 nM MTX3 increased NR2A/B levels, and effect opposite to that previously described for CTX3C in mice cortical neurons. The number of independent determinations is shown in parenthesis. ** *p* < 0.01 versus control.

The most abundant excitatory neurotransmitter receptors in the brain belong to the ionotropic class of glutamate receptors, which mediate fast synaptic transmission and readily adapt to counteract changes in neuronal activity [35,36]. Since activation of voltage-gated sodium channels by CTX3C and the consequent change in neuronal activity has been shown to regulate inhibition and excitation in opposite directions in mice cortical neurons [25], next, we evaluated the effect of gambierone and MTX3 on ionotropic glutamate receptors expression. In this sense, we have previously reported that chronic exposure of primary cortical neurons to CTX3C elicited long term alterations in synaptic neurotransmission leading to the down-regulation in the protein level of ionotropic glutamate receptors [37]. Therefore, in order to compare the long term effects of gambierone and MTX3 to those of CTX3C, differentiated CTX0E16 neurons were maintained in the presence of 20 nM gambierone or 20 nM MTX3 for 15 days in culture with full toxin renewal each two days, and the expression of both α-amino-3-hydroxy-5-methyl-4-isoxazolepropionic acid (AMPA) and N-methyl-d-aspartate (NMDA) receptor subunits was evaluated. As shown in Figure 5A, exposure of human neurons to gambierone or MTX3 decreased the level of the AMPA receptor subunits by 21.31 ± 7.47% (*n* = 12, *p* = 0.0093) and 23.06 ± 8.17% (*n* = 12, *p* = 0.009), respectively. However, both compounds increased the expression of the NMDA receptors subunits by 83.45 ± 24.51% (*n* = 9; *p* = 0.0036) and 87.11 ± 26.26 (*n* = 7; *p* = 0.0051) respectively (Figure 5B). Thus, even when the activity of gambierone and MTX3 seems similar to the biological activity of CTX3C in primary mice cortical neurons [37] both compounds increased the protein expression of NMDA receptors while chronic CTX3C decreased the expression of both NMDA and AMPA receptor subunits [37]. 

**Figure 6:**
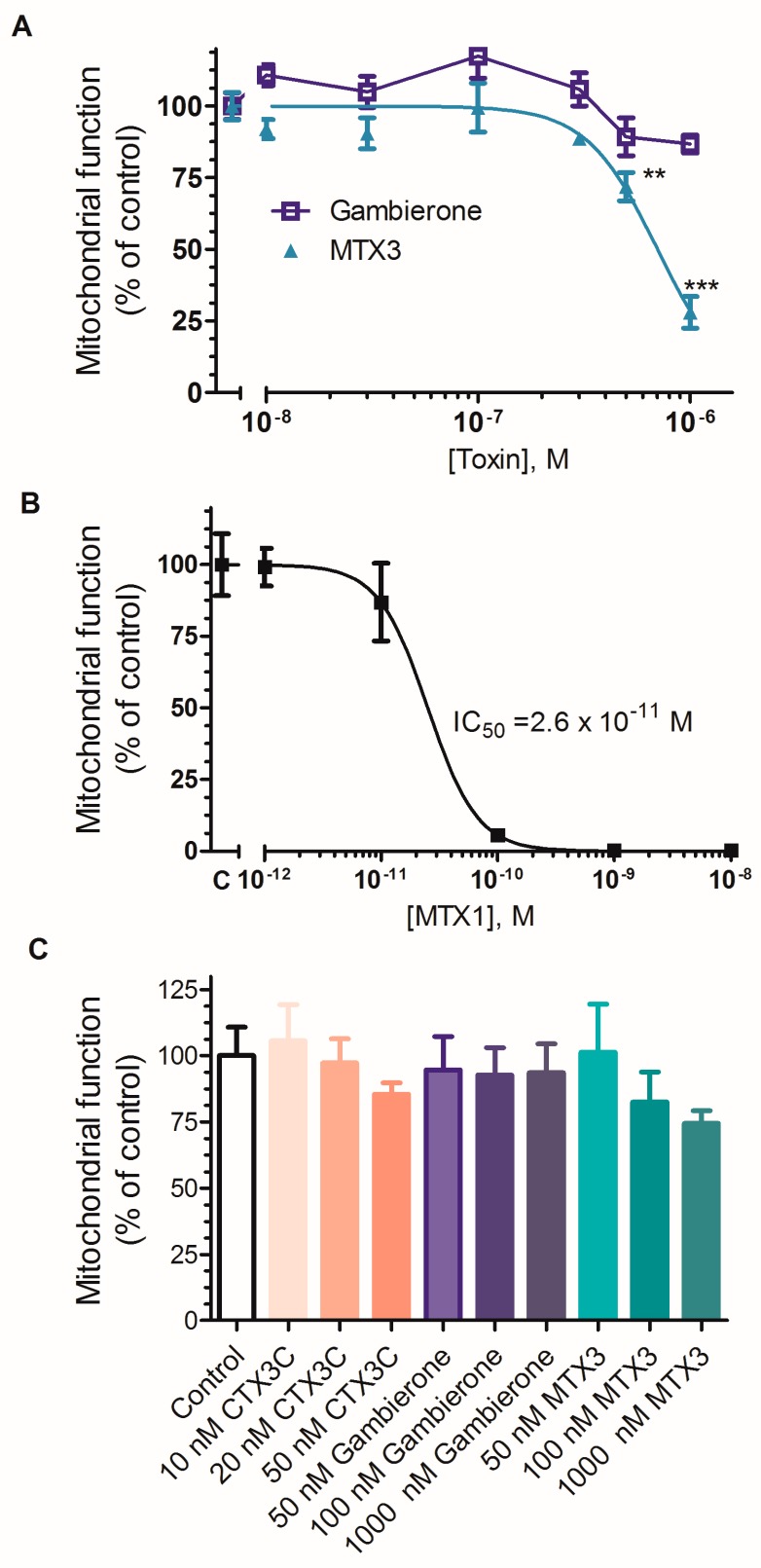
Effect of high concentrations of gambierone and MTX3 on cell viability in undifferentiated human neuroblastoma cells. (**A**) Four days exposure of human neuroblastoma cells to either gambierone or MTX3 differentially affected cell viability. At concentrations of 100 and 1000 nM MTX3 significantly decreased cell viability while gambierone, at the same concentrations, did not alter mitochondrial function. (**B**) 24 hours exposure of neuroblastoma cells to MTX1 evoked complete cell death at 0.1 nM. (**C**) Exposure of neuroblastoma cells to synthetic ciguatoxin CTX3C, gambierone or MTX3 for 24 hours did not affect cell viability. Data are mean ± sem of 3 to 4 independent experiments, each performed in triplicate wells. ** *p* < 0.01; *** *p* < 0.005 vs. control.

In view of the subtle differences between the chronic neuronal alterations produced by CTX3C in mice cortical neurons [37] and those produced by gambierone and MTX3 reported here the effects of both gambierone and MTX3 on cell viability were additionally evaluated at higher concentrations in human neuroblastoma cells since this cellular model was initially employed to assed the biological effects of gambierone [19]. Cell viability was evaluated using the MTT assay which evaluates the ability of the nicotinamide-adenine-dinucleotide (NAD(P)H) coenzyme and dehydrogenases from metabolically active cells to reduce tetrazolium salts and form strongly colored and lipophilic formazan products which are then quantified by absorbance or fluorescence [38]. The amount of colored formazan product is in linear correlation with a healthy mitochondrial function and with the cell viability [39]. As indicated in Figure 6, exposure of undifferentiated human neuroblastoma cells for 4 days to either gambierone or MTX3 at concentrations ranging from 10 to 1000 nM led to different effects on cell viability (Figure 6A). As expected from our previous work [19] gambierone did not cause cell death even at concentrations of 1 µM while MTX3 at the highest concentration evaluated decreased cell viability by more than 70% with an IC_50_ of 7.02 × 10^−7^ M (95% confidence intervals from 6.09 × 10^−7^ to 8.1 × 10^−7^ M). In contrast, 24 hours exposure of human neuroblastoma cells to MTX1 caused complete cell death at concentrations as low as 0.01 nM (Figure 6B) while neither CTX3C, nor gambierone or MTX3 affected cell viability after 24 hours (Figure 6C). Thus, these results provide additional data indicating that MTX3 exhibits ciguatoxin-like activity rather than a maitotoxin-like toxicity.

## 3. Discussion

Ciguatera is a human foodborne disease caused by the consumption of fishes that have accumulated toxins produced by dinoflagellate organisms of the genera *Gambiersdiscus* and *Fukuyoa* [11,12,29]. These dinoflagellates produce lipophilic toxins known as ciguatoxins and water soluble maitotoxins. Most *Gambiersdiscus* and *Fukuyoa* strains produce maitotoxins while only some of them produce ciguatoxins [12]. Symptoms of ciguatera fish poisoning in humans include gastrointestinal disturbances (diarrhea and vomiting), cardiac alterations with hypotension and bradycardia as well as neurological problems manifested mainly as paresthesiae and peripheral neuropathy and rarely death [31,40]. Presently, ciguatera is considered one of the most common non-bacterial food borne diseases, affecting 10,000 to 500,000 people worldwide each year [4]. Although traditionally the presence of *Gambierdiscus* was reported mainly in tropical areas, ciguatera intoxications have been identified recently in Europe and are common in the Canary Islands and Madeira [6,7,8,41]. Up to date four analogs of MTX have been identified from different strains of these dinoflagellates albeit, so far, the chemical structure was solved only for the MTX1 and MTX4 analogs [10,12,13]. Although compounds p-MTX2 and p-MTX3 were recently detected in several species of *Gambierdiscus* from the Canary Islands, their structure identification was not possible because of the lack of reference material and the low amount of metabolites isolated [10,13]. In fact, previous reports highlighted that since the specific molecular target of MTX is still unknown its structure-activity relationship can only be inferred from NMR structural features and analogies with other ladder-shaped polyether toxins [13,19]. In contrast to the known activity of CTX3C activating neuronal sodium channels at low nanomolar concentrations without evoking neuronal toxicity [19,25] the only report evaluating the biological activity of gambierone reported that this compound was less active on sodium channels [19] while MTX1 caused rapid neuronal death due to a high and fast intracellular calcium load [33]. However, several pathways for the MTX1-induced calcium influx have been proposed including nonselective, non-voltage-activated ion channels, L type calcium channels and transient receptor potential channels [33,42,43]. MTX3 was first reported from cultures of an Australian clone WC1/1 of *Gambierdiscus toxicus* in 1994 [10]. Since then, neither the chemical structure nor the bioactivity of MTX3 have been described. This was mainly due to the difficulty to scale up the cultures of *Gambierdiscus* spp. which yielded scarce amounts of MTX3 and therefore prevented any assessment of its bioactivity and the use of NMR to elucidate its structure. For about two decades, it has been assumed that MTX3 contained two sulfate esters through a putative assignment of the peaks generated from an Ion Spray mass spectrometer in positive mode [29]. The presence of at least one sulfate was demonstrated by desulfation experiments and it was demonstrated to be responsible for the bioactivity of the MTX congeners [44]. While comparing the MS data of compound **1** to those of p-MTX3, a very similar fragmentation pattern was highlighted. Providing the NMR data of **1** we are highly confident that MTX3 and **1** are in fact the same compound, clarifying thereby the mystery surrounding the structure of MTX3 (Figures S1–S8). The structural informative fragments described here will be very useful from a screening point of view, particularly in high throughput screenings using selected ion monitoring or selected reaction monitoring. 

After the chemical identification of MTX3, its biological activity was evaluated using well characterized cellular models [19,32]. Presently, it was demonstrated that the parent compound MTX1 modulated different ion channels and altered many cellular functions even if its mechanism of action is still controversial. For instance, we and others have previously described that the toxin induces a rapid and sustained rise in the cytosolic calcium concentration in several cellular models, and this effect, in conjunction with the cellular acidification induced by this toxin could led to a rapid cell death [22]. However the exact cellular target of this toxin is yet unknown and several options including nonselective cation channels [8,20], voltage-gated calcium channels [45] and TRP channels [21,33] have been investigated. In contrast, ciguatoxins are potent activators of voltage-gated sodium channels and some of the analogs (P-CTX-1B), but not all of them, have a modest effect on the levels of cytosolic calcium. In this sense, the initial characterization of the biological activity of gambierone reported a small rise in the cytosolic calcium concentration at nanomolar concentrations [19] however its cytotoxicity was not previously evaluated. Here, we confirmed and extended the study of the biological activity of gambierone in cultured human cortical neurons and compared it with the bioactivity of the related new chemical entity. As reported here both gambierone and 44-methylgambierone (MTX3) shared a similar biological activity characterized by the lack of cytoxicity at nanomolar concentrations and a small effect on the cytosolic calcium concentration that was similar to that previously described for the P-CTX-like activity [27] in contrast to the known huge increase in calcium elicited by MTX1 in different cellular models [22,27,33,45]. However, at higher concentrations MTX3, in contrast to gambierone, caused a decrease in cell viability in undifferentiated neuroblastoma cells opening the door to further explore its putative therapeutic effects (for example in glioma treatment). In fact, the marine compounds crambescidins induced moderate rises in neuronal cytosolic calcium through activation of ionotropic glutamate receptors [46] and have a profitable activity inhibiting tumor growth [47]. Interestingly, chronic exposure of human neurons to either gambierone or MTX3 modified the expression of excitatory neurotransmiter receptor subunits, an alteration that was observed also after exposure of mice cortical neurons to CTX3C [37] and that could be involved in the neurological symptoms caused by ciguatera in humans [4]. Altogether and in addition to the structure elucidation of MTX3, the data presented here confirm the similarity between the biological activities of gambierone, MTX3 and CTX3C. In this sense, we have previously associated common neurological alterations in patients suffering from ciguatera food poisoning such as fatigue, weakness, depression, and memory loss [4] with alterations in synaptic transmission as a consequence of decreased excitatory activity that was attributed in part to the decrease in the protein level of ionotropic glutamate receptors [37]. Therefore, the neuroactivity of both gambierone and MTX3 should be further explored and quantified to establish their contribution to the long-lasting neurological alterations caused by ciguatera in humans.

## 4. Materials and Methods 

### 4.1. General Experimental Procedures

^1^H and 2D NMR experiments were recorded on a Varian Inova 750 MHz NMR spectrometer. ^13^C NMR spectra were recorded at 125 MHz in a Varian VNMRS-500-WB instrument (Agilent Technologies, Palo Alto, CA, USA). Chemical shifts (δ in ppm) are referenced to the carbon (δ_C_ 49.0) and residual proton (δ_H_ 3.31) signals of CD_3_OD. Mass spectra were obtained from a Xevo TQ mass spectrometer (Waters, Manchester, UK) connected to an ACQUITY Ultra High Performance Liquid Chromatography (UPLC) system (Waters, Manchester, UK). Purification was performed on a Waters HPLC system: Waters 2767 sample manager, Waters system fluidics organizer, Waters 2545 binary solvent manager, Waters 515 post-column HPLC pump, Waters 3100 mass detector. HRMS was acquired by Ion Trap-Time of Flight (IT-TOF) mass spectrometer instrument from Shimadzu (Kyoto, Japan)

### 4.2. Biological Material

The strain CCMP401 of Gambierdiscus belizeanus was obtained from The Provasoil-Guillard National Center for Marine Algae and Microbiota (NCMA). 

### 4.3. Cell Culture

The CCMP401 strains were cultured and grown in seawater enriched with modified K medium free of silicates [1.764 mM NaNO_3_, 0.01 mM Na-β-glycerophosphate, 0.01 nM H_2_SeO_3_, 1 mM Tris-Base, 0.11 mM Na_2_EDTA, 0.01 mM FeCl_3_-6H_2_O, 0.9 µM MnCl_2_-4H_2_O, 0.08 µM ZnSO_4_-7H_2_O, 0.05 µM CoCl_2_-6H_2_O, 0.026 µM Na_2_MoO_4_-2H_2_O, 0.296 µM thiamine (vit B1), 2.05 nM biotin and 0.369 nM cyanocobalamin (Vit B12)] as previously described [19,48,49]. Briefly, the culture medium was natural seawater, at 33 salinity level, 24 °C and a photoperiod of 14 hours light/10 hours dark, conditions that favored the growth of the cultures. The cellular growth was divided in steps, increasing the volume progressively to reach 20 L bags cultures.

### 4.4. Extraction and Purification

#### 4.4.1. Extraction

Large scale culture of G. belizeanus CCMP401 was grown in a bag of 20 L for a total culture size of 2000 L. Cells were harvested by filtration with a 20 μm mesh and suspended in methanol (MeOH). Then the cells were lyzed by ultrasound (3 cycles) and stored at −20 °C. The methanolic extracts were centrifuged and the pellets were re-extracted (x2) with MeOH.

#### 4.4.2. Separation and Purification

The combined methanolic extracts were subjected to evaporation and after dilution with water, the aqueous solution was extracted first with diethyl ether twice. Diethyl ether extract was dried and dissolved in MeOH 90%, a new step of liquid-liquid extraction with MeOH 90%: Hexane was performed twice. Methanolic residues obtained after evaporation were dissolved in ethyl acetate:MeOH (9:1) and subjected to further purification by column chromatography:

- Florisil low-pressure column, 45 × 4.5 cm pre-equilibrated with EtOAc/MeOH (9:1). The column was successively eluted with EtOAc/MeOH (9:1, 500 mL), EtOAc/MeOH (8:2, 500 mL), EtOAc/MeOH (7:3, 500 mL) and finally MeOH (500 mL). Ethyl acetate fractions were dried and dissolved in chloroform for the next column.
- Silica low-pressure column, 45 × 4.5 cm pre-equilibrated with CHCl_3_. The column was successively eluted with CHCl_3_ (500 mL), CHCl_3_/MeOH (9:1, 500 mL), CHCl_3_/MeOH (8:2, 500 mL), CHCl_3_/MeOH (7:3, 500 mL) and finally MeOH (500 mL). Combined chloroform extracts were evaporated and dissolved in CHCl_3_/MeOH 2:1.
- Sephadex LH20 low-pressure column, 60 × 2.5 cm pre-equilibrated with CHCl_3_/MeOH (2:1). Toxins were eluted from 60 mL to 100 mL of CHCl_3_/MeOH (2:1) with fractions every 3 mL.
- Sephadex LH20 low-pressure column, 60 × 2.5 cm pre-equilibrated with MeOH. Toxins were eluted from 60 mL to 100 mL of MeOH with fractions every 3 mL.
- C18 low-pressure column, 60 × 2.5 cm pre-equilibrated with 50 % MeOH and eluted with a stepwise gradient of 50, 60, 70, 80, 90 and 100% of MeOH (150 mL each with fractions every 15 mL). Gambierone eluted from fraction 28 to 43 and MTX3 from 32 to 43. Fractions 28–43 were then combined.
- C18 low-pressure column, 60 × 2.5 cm pre-equilibrated with 35% MeCN and eluted successively with 35, 40, 45, 50 and finally 60% MeCN (150 mL each using 15 mL fractions). Gambierone was eluted in fractions 13 to 17 and MTX3 from 18 to 21.

Each toxin fraction was separately combined and applied to a preparative HPLC method. The HPLC system, from Waters, consists of Binary Gradient Module, M515 HPLC Make-up Pump, autoinjector and collector 2767 Sample Manager and System Fluidics Organizer. This system is coupled to a MS detector, 3100 Mass Detector. The column used was X-Select Phenyl-Hexyl (10 × 250 mm, 5 μm) from Waters, with a flow rate of 6 mL/min and 350 μL of injection volume. The chromatographic conditions are as follows, isocratic H_2_O:MeOH (40:60) for 25 min followed by a gradient to H_2_O:MeOH (2:98) in 25 minutes, hold for 10 minutes and finally a re-equilibration to initial conditions. Elution and collection was performed with a splitter 1:100 with a MS solution which consists in (MeOH 62.5%, 1mM AcONH4). Peak detection was carried out using ESI and select ion monitoring (SIM) acquisition. 15 mg of gambierone (**2**) and 6 mg of MTX3 (**1**) were obtained. 

### 4.5. Mass Spectrometry

Gambierone and MTX3 analyses were performed on an ACQUITY UPLC system coupled with Xevo TQ MS mass spectrometer from Waters (Manchester, UK). Chromatographic fraction identification was achieved in AQUITY UPLC BEH C18 (2.1 × 100 mm, 1.7 μm, Waters) column equipped with a 0.2 μm ACQUITY UPLC in-line filter. The composition of the mobile phase was water (A) and ACN/water (95:5) (B), both buffered with 50 mM formic acid and 2 mM ammonium formate. The mobile phase flow rate was 0.4 mL/min, and the injection volume was 5 μL in methanol. Chromatographic separation was performed by gradient elution, starting with 35% B increasing to 100% B in 5 min, then 100% B is held for 5 min and reducing afterward to 35% for 0.1 min; this proportion was maintained for 1.9 min yielding MTX3 at 3.15 min and gambierone at 2.90 min. Non-methanolic fractions were dried down and reconstituted to analyze each sample. Analysis was carried out using positive electrospray ionization (ESI+) and SIM acquisition.

Xevo TQ MS mass spectrometer was operated with the following optimized source-dependent parameters: capillary potential (V) 2.5 kV, desolvation gas flow 850 L/h N_2_, desolvation temperature 350 °C, cone gas flow 50 L/h N_2_, collision gas flow 0.12 mL/min, source temperature 150 °C, cone voltage 30 V, collision gas Ar at 4.5 103 mbar, extractor 3 V. Data were acquired using Waters MassLynx software and processed using the TargetLynx Application Manager.

#### High Resolution Mass Spectrometry

A UPLC system with an IT-TOF mass spectrometer instrument from Shimadzu (Kyoto, Japan) was used. The UPLC instrument consisted of a system controller (CBM-20A), a column oven (CTO-10AS), an autoinjector with a refrigerated rack (SIL-10AC), degasses (DGU-20A 5R) and two pumps (LC-30AD). The chromatographic separation was performance with an AQUITY UPLC BEH C18 column (2.1 × 100 mm, 1.7 µm particle size, Waters Spain) at 35 °C and a flow rate of 0.4 mL/min. Mobile phase A was water with 50 mM formic acid and 2 mM ammonium formate and mobile phase B was acetonitrile:water (95:5) containing 50 mM formic acid and 2 mM ammonium formate. The gradient program for chromatographic separation was started with 50% A for 2.41 min, then decreasing to 0% A within 4.53 min, and was held for 4.54 min. Finally, the initial condition was returned in 0.52 min and this condition, 50% A, was maintained 2 min until the next injection.

The mass spectrometer was equipped with an ESI source and it was operated with the following conditions: a heat block and curve desolvation line temperature, 200 °C; nebulizing gas flow, 1.5 L/min; detector voltage, 1.65 kV; and drying gas pressure, 105 kPa. The mass spectra were acquired in full scan MS mode in positive and negative mode with a mass range of 50-150 and 150–2000 Da. The molecules were analyzed using an ion accumulation time was set to 30 ms, with an event time of 300 ms and 3 repetitions. The mass range was calibrated prior to data acquisition employing a direct infusion of IT-TOF standard sample.

### 4.6. Evaluation of the Biological Activities

#### 4.6.1. Toxins and Drugs Used

MTX1 (purity higher than 99%) and CTX3C were obtained from Wako (FUJIFILM Wako Chemicals Europe GmbH, Neuss, Germany) and dissolved at a concentration of 10 μM in DMSO. Toxin dilutions were performed in deionized water or Locke’s buffer solution. Gambierone was from CIFGA (Lugo, Spain), the stock solution was 157.4 µM and was diluted to 10 µM in DMSO. MTX3 was also from CIFGA and working solutions were prepared from a 10 µM stock in DMSO that was obtained from the initial 164.4 µM stock. All other chemicals were of reagent grade and purchased from Sigma or Tocris.

#### 4.6.2. Cortical Human Neural Stem Cell Line CTX0E16 Culture and Differentiation

The neuronal stem cell line CTX0E16 was immortalized from the cerebral cortex of a 12 weeks of gestation fetus, by the ectopic expression of the c-mycER^TAM^ transgene and kindly provided by a material transfer agreement with ReNeuron Limited (Guildford, Surrey, UK). CTX0E16 cells were cultured following the provider instructions as previously reported [32,50]. Briefly, in proliferative conditions cells were seeded onto Poly-D-lysine (PDL, 5 μg/cm^2^, Sigma, cMerck KGaA, Darmstadt, Germany) and laminin-coated (1 μg/cm^2^; Sigma) tissue culture flasks and maintained in DMEM:F12 medium supplemented with 15 mM HEPES and sodium bicarbonate (Sigma), 0.03% human serum albumin (Sigma), 100 μg/ml apotransferrin (Scipac Ltd , Kent, UK), 16.2 μg/ml putrescine (Sigma), 5 μg/ml human insulin (Sigma), 60 ng/ml progesterone (Sigma), 2 mM L-glutamine (Sigma) and 40 ng/ml of sodium selenite (Sigma), 10 ng/ml of human fibroblast growth factor (FGF2, PeproTech, Rocky Hill, NJ, USA), 20 ng/ml of human epidermal growth factor (EGF, PeproTech, Rocky Hill, NJ, USA) and 100 nM hydroxy tamoxifen (4-OHT, Sigma). Full media changes were performed every 2–3 days and cells were passaged after reaching 70–80% confluence using accutase (Sigma) and maintained between 25 and 30 passages. All the experiments were carried out using cells from passages 12 to 30. CTX0E16 cultures were differentiated in Neurobasal Medium (Thermofisher) supplemented with human serum albumin, apotransferrin, putrescine, human insulin, progesterone, L-glutamine, and sodium selenite at the concentrations used for proliferation and 1X B27 serum-free supplement (Thermo Fisher, Thermo Fisher Scientific, Madrid, Spain) with half medium changes every 2–3 days. Cultures were differentiated for up to 40 days.

#### 4.6.3. Neuroblastoma Cell Line 

Neuroblastoma cell line SH-SY5Y was from American Type Culture Collection (ATCC) Number CRL-2266. The cells were seeded in 25-cm^2^ culture flasks and maintained in Eagle’s Minimum Essential Medium (EMEM) from ATCC and F12 Medium (Invitrogen) 1:1 supplemented with 10% fetal bovine serum (Gibco, Thermo Fisher Scientific, Madrid, Spain), 1% GlutaMAX (Gibco) and antibiotics (100 UI/mL penicillin and 100 µg/mL streptomycin). 

#### 4.6.4. Determination of Cellular Viability

Cell viability was assessed by the MTT (3-(4,5-dimethylthiazol-2-yl)-2,5-diphenyltetrazolium bromide) test. For viability assays cells were treated with the toxins for the indicated periods in culture medium. After treatment, cells were incubated with an MTT solution at a final concentration of 500 µg/ml in Locke’s buffer containing (in mM): 154 NaCl, 5.6 KCl, 1.3 CaCl_2_, 1 MgCl_2_, 10 HEPES, and 5.6 glucose (pH 7.4) for 1 hour at 37 °C. Following washing off excess MTT, cells were disaggregated with 5% sodium dodecyl sulfate overnight, and cell viability was determined measuring the absorbance of the colored formazan salt at 595 nm in a spectrophotometer plate reader. Saponin was used as death control.

#### 4.6.5. Determination of Cytosolic Calcium Concentration ([Ca^2+^]_c_)

Mature neurons seeded onto 18 mm glass coverslips were loaded for 30 min at 37 °C with the cell permeant calcium sensitive dye Fura-2 acetoxymethyl ester (Fura-2 AM), dissolved in Locke’s buffer at a final concentration of 2.5 μM [33]. After incubation, loaded cells were washed three times with cold Locke’s buffer. Cytosolic calcium was measured with a Nikon Diaphot 200 microscope with epifluorescence optics (Nikon 40×-immersion UV-Fluor objective, (Izasa Scientific S.L.U., Barcelona, Spain)) and fluorescence equipment Lambda-DG4 (Sutter Instruments, Izasa Scientific S.L.U., Barcelona, Spain ). The light source was a xenon arc bulb and the excitation wavelengths were selected with filters. Excitation wavelengths were 340 and 380 nm, and the emission was collected at 510 nm [46]. Data were analyzed with the Metafluor software (version 7.1, Molecular Devices, LLC, Sunnyvale, CA, USA) and expressed as the 340/380 fluorescence ratio. In all the cases, each individual experiment represents the mean of the cells from duplicate coverslips and data were obtained from at least three independent cultures. 

#### 4.6.6. Western Blot

The protein level of glutamate receptors was evaluated in cultures of mature human cortical neurons maintained for 15 days in the presence of gambierone or MTX3, both at 20 nM, with half medium changes and toxin replacement each two days. After treatment the cells were washed three times with cold PBS and cell lysates were obtained using RIPA lysis buffer (Thermofisher), containing 25 mM Tris-HCl (pH 7.6), 150 mM NaCl, 1% NP-40 (nonyl phenoxypoliethoxylethanol), 1% sodium deoxycholate and 0.1% SDS supplemented with commercial phosphatase and protease inhibitors cocktails (Thermofisher), and stored at −20 °C when needed. Total protein concentration in cell lysates was measured in triplicate by the Bradford assay, using bovine serum albumin (BSA) as standard. For SDS-PAGE electrophoresis cell lysates samples containing 30 μg of total protein were denaturalized in commercial 4× Laemmli sample buffer (Bio-Rad, containing 277.8 mM Tris-HCl, pH 6.8, 4.4% SDS, 44.4% glycerol, and 0.02% bromophenol blue, supplemented with 2.5% 2-mercaptoethanol (Bio-Rad Laboratories S.A., Barcelona, Spain)) and resolved in 10% polyacrylamide gels at a constant voltage of 200 V for 38 minutes. Afterwards, proteins were transferred to PVDF membranes (10 V for 30 min) using a semi-dry transfer cell (Bio-Rad). The membranes were blocked for 1 hour with 5% BSA and incubated overnight at 4 °C with primary polyclonal antibodies against AMPA receptor subunits (GluR2,3,4 at a dilution of 1:500,) or NMDA receptor subunits 2A/B (1:500) both from Thermofisher. Protein bands were detected using the Supersignal West Pico chemiluminescent substrate (Pierce, Thermo Fisher Scientific, Madrid, Spain) and the Diversity 4 gel documentation and analysis system (Syngene, Cambridge, UK). Chemiluminescence was quantified with the Diversity GeneSnap software (Syngene). β-actin (Millipore, Merck Chemicals & Life Science S.A., Madrid, Spain) was used as control for lane loading and to normalize chemiluminescence values. Each condition was analyzed in duplicate wells for each treatment. 

#### 4.6.7. Statistical Analysis

All data are expressed as means ± SEM of n determinations. Statistical comparison was by Student’s *t* test. *p* values < 0.05 were considered statistically significant.

## Figures and Tables

**Table 1:** ^1^H (750 MHz) and ^13^C (125 MHz) NMR Data for **1** in CD_3_OD.

Atom Number	*δ*_H_, Mult. (*J* in Hz)	*δ*_C_	n°	*δ*_H_, Mult. (*J* in Hz)	*δ*_C_
1	3.47, dd (11.0, 4.5) 3.42, m	67.8	27	3.51, m	77.7
2	4.10, m	69.8	28	2.19, m 1.33, m	39.8
3	2.00, m 1.71, dd (14.5, 10.0)	39.7	29	3.12, m	70.1
4	-	100.7	30	2.94, m	78.1
5	4.21, d (3.0)	73.0	31	1.89, m 1.54, t (11.5)	34.9
6	4.70, dd (10.0, 3.0)	77.8	32	3.76, dd (8.5, 3.5)	72.5
7	3.37, m	77.3	33	-	77.3
8	3.77, m	67.9	34	2.34, d (12.5) 2.15, d (12.5)	54.1
9	2.17, m 1.58, t (11.5)	37.9	35	-	143.5
10	3.35, m	79.6	36	2.54, d (14.5) 2.21, d (14.5)	43.3
11	3.79, m	82.4	37	-	79.9
12	5.64, dt (12.5, 2.0)	132.8	38	4.06, dt (10.5, 3.0)	73.1
13	5.75, dt (12.5, 2.0)	133.0	39	2.61, m 2.59, m	45.8
14	3.81, m	83.0	40	-	211.6
15	3.44, dd (5.0, 2.0)	79.9	41	2.59, m	43.9
16	1.99, m 1.49, t (12.0)	47.4	42	2.39, q (7.0)	23.3
17	-	76.6	43	5.45, t (7.5)	132.3
18	3.01, dt (11.0, 2.5)	86.8	44	-	136.0
19	1.79, m 1.62, m	25.4	45	6.33, dt (17.5, 10.5)	142.6
20	1.95, m 1.80, m	34.2	46	5.09, d (17.5) 4.90, d (11.0)	111.2
21	3.53, m	87.4	47	1.20, s	16.4
22	3.54, m	75.7	48	1.00, d (7.5)	13.5
23	1.82, m 1.64, m	32.8	49	1.19, s	16.9
24	1.92, m 1.78, m	29.5	50	4.98, br s 4.85 ^a^	118.6
25	2.19, m	35.6	51	1.13, s	20.7
26	3.11, m	85.9	52	1.74, s	11.7

^a^ Overlapped with HOD. d: doublet, m: multiplet, t: triplet, s: singlet, br: broad.

**Table 2:** Comparison between ions and fragments in positive mode for **1** and MTX3 in the literature. nd: not detected.

Peaks	Ion Species and Fragments Reported in ESI(+)-LRMS for:	
	Compound 1	MTX3 by Lewis et al. [29]
1099.5	nd	[M-H+2Na+K]^+^
1083.5	[M-H+2Na]^+^	[M-2H+3Na]^+^
1077.5	[M+K]^+^	[M-H+Na+K]^+^
1061.5	[M+Na]^+^	[M-H+2Na]^+^
1056.5	[M+NH_4_]^+^	nd
1055.5	nd	[M+K]^+^
1039.5	[M+H]^+^	[M+Na]^+^
1021.5	[M+H-H_2_O]^+^	[M+Na-H_2_O]^+^
1003.5	[M+H-2H_2_O]^+^	[M+Na-2H_2_O]^+^
996.5	nd	[M-H+Na+K-SO_3_]^+^
981.5	[M+Na-SO_3_]^+^	[M-H+2Na-SO_3_]^+^
963.5	nd	[M-H+2Na-H_2_O-SO_3_]^+^
959.5	[M+H-SO_3_]^+^	[M+Na-SO_3_]^+^
941.5	[M+H-H_2_SO_4_]^+^	[M+Na-H_2_O-SO_3_]^+^
923.5	[M+H-H_2_O-H_2_SO_4_]^+^	[M+Na-2H_2_O-SO_3_]^+^
905.5	[M+H-3H_2_O-SO_3_]^+^	[M+Na-3H_2_O-SO_3_]^+^
887.5	[M+H-4H_2_O-SO_3_]^+^	[M+Na-4H_2_O-SO_3_]^+^

nd: not determined.

**Table 3:** Optimized MS/MS spectrometric parameters for the MRM transitions.

Compound	Maitotoxin-3				Gambierone			
ESI Mode	Parent Ion	Cone/V	Fragment Ion	Coll/ev	Parent Ion	Cone/V	Fragment Ion	Coll/ev
+	1039.5	30	233.1	30	1025.5	30	219.1	30
			277.1	60			277.1	60
			803.4	30			803.4	30
			941.5	30			927.5	30
			1021.5	4			1007.5	4
−	899.6	70	96.8	60	899.6	70	96.8	60
	1037.5	70	96.8	60	1023.5	70	96.8	60
			899.6	60			899.6	60

## Supplementary Materials

The following data are available online at http://www.mdpi.com/2072-6651/11/2/79/s1, Figure S1: ^1^H NMR spectrum of **1** at 750 MHz in CD_3_OD. Figure S2: ^13^C NMR spectrum of 1 at 125 MHz in CD_3_OD. Figure S3: DEPT135 NMR spectrum of 1 at 125 MHz in CD_3_OD. Figure S4: COSY NMR spectrum of 1 at 750 MHz in CD_3_OD. Figure S5: zTOCSY NMR spectrum of **1** at 750 MHz in CD_3_OD. Figure S6: CRISIS-HSQC NMR spectrum of **1** at 750 MHz in CD_3_OD. Figure S7: HMBC spectrum of 1 at 750 MHz in CD_3_OD. Figure S8: ROESY NMR spectrum of 1 at 750 MHz in CD_3_OD.
