# Supplementary material for: Structure Elucidation and Biological Evaluation of Maitotoxin-3, a Homologue of Gambierone, from Gambierdiscus belizeanus
Source: Toxins (Basel). 2019 Feb 1;11(2):79. doi: 10.3390/toxins11020079 (PMC6409949; doi:10.3390/toxins11020079)
Supplement: Supplementary file 1 [file toxins-11-00079-s001.pdf]

Supplementary Materials: Structure Elucidation and Biological Evaluation of Maitotoxin-3, a Homologue of Gambierone, from *Gambierdiscus belizeanus*

Andrea Boente-Juncal, Mercedes Álvarez, Álvaro Antelo, Inés Rodríguez, Kevin Calabro, Carmen Vale, Olivier P. Thomas and Luis M. Botana

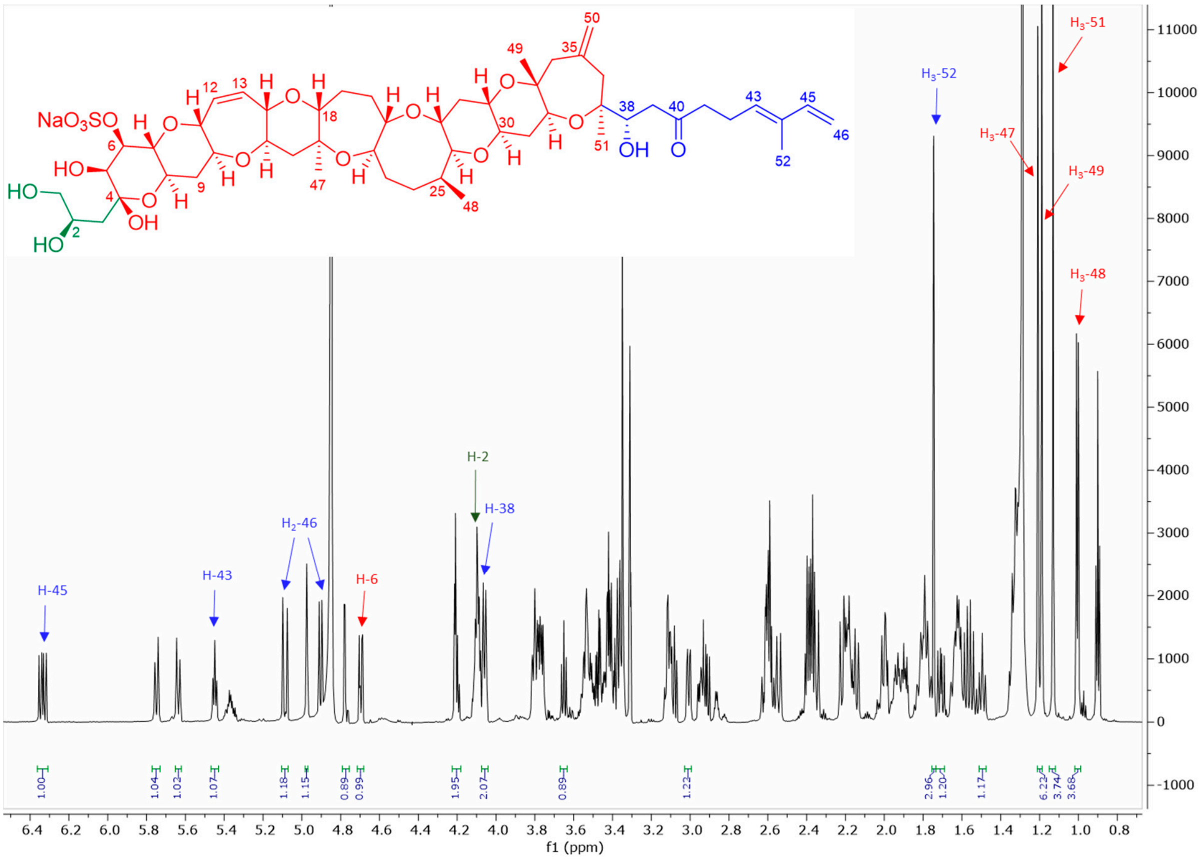

Figure S1. ^{1}H NMR spectrum of **1** at 750 MHz in CD_3OD.

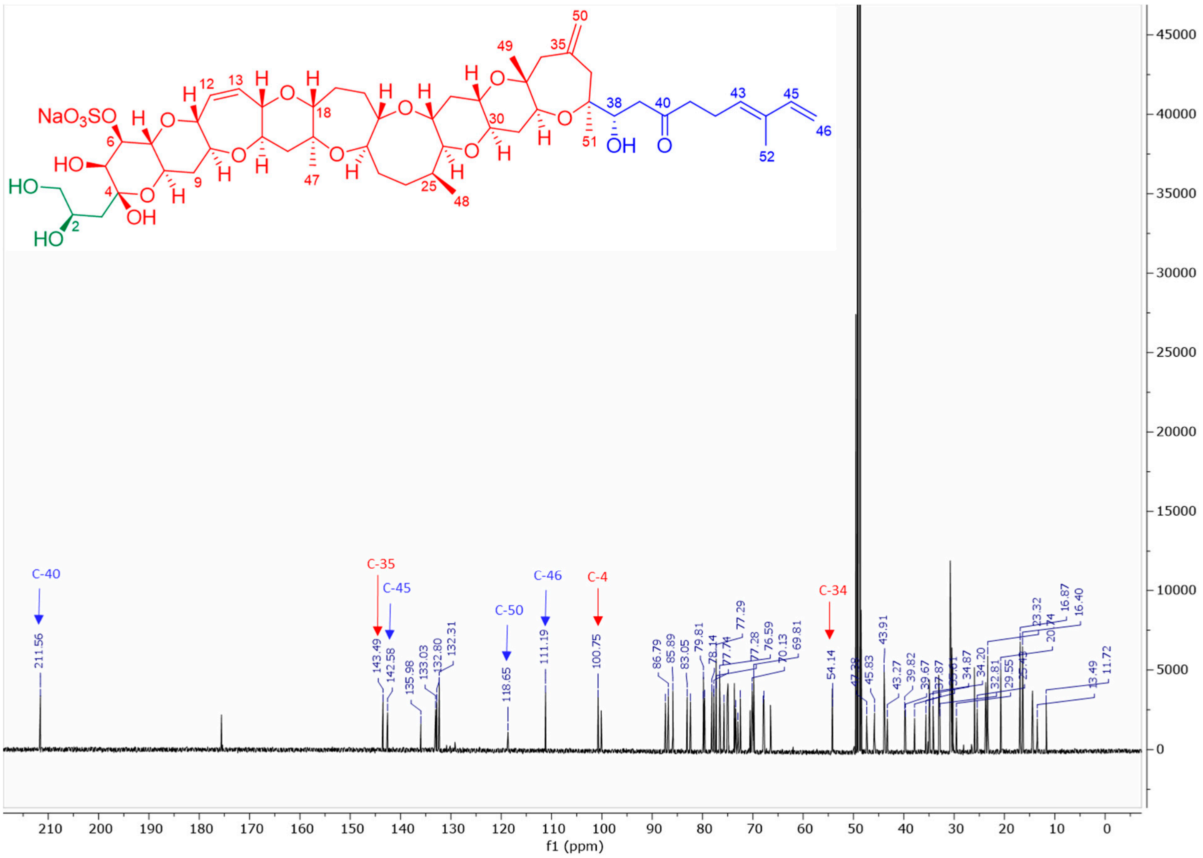

Figure 2. ^{13}C NMR spectrum of 1 at 125 MHz in CD_3OD.

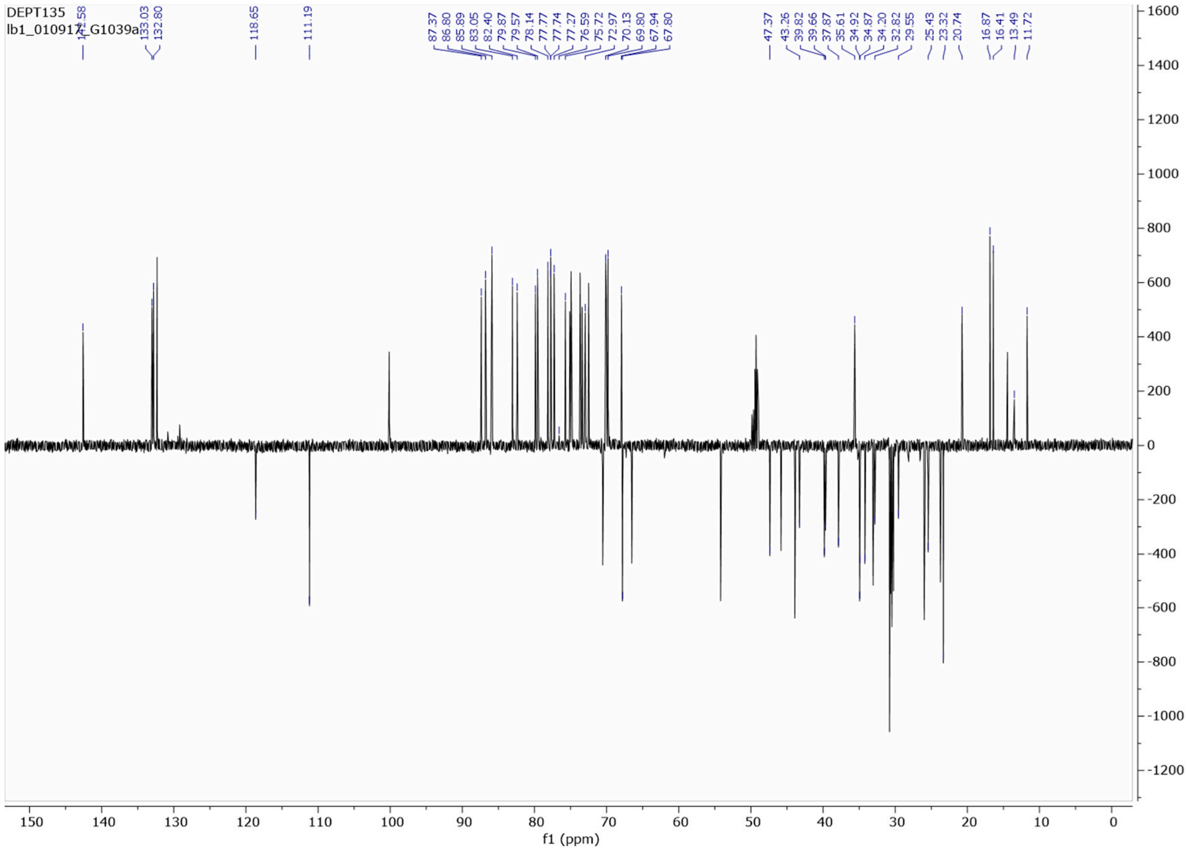

Figure 3. DEPT135 NMR spectrum of 1 at 125 MHz in CD_3OD.

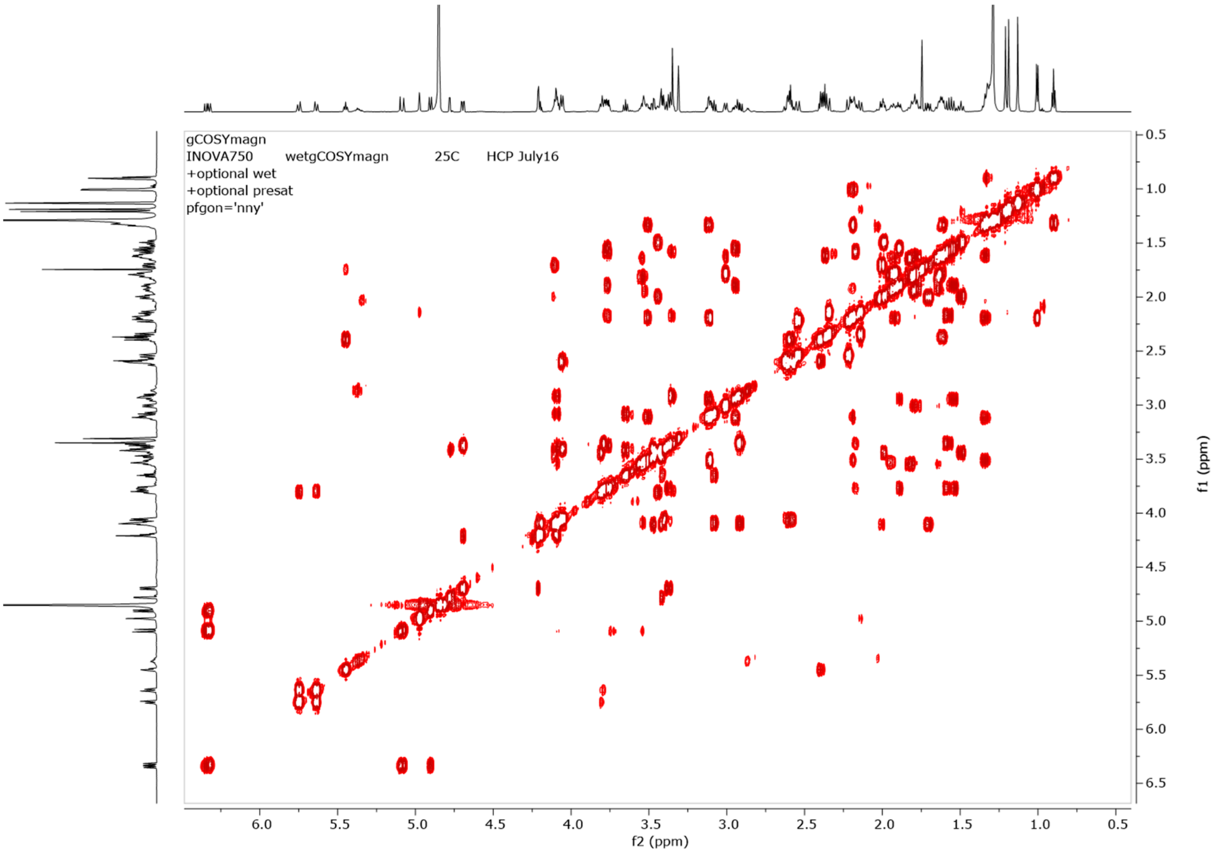

Figure 4. COSY NMR spectrum of 1 at 750 MHz in CD_3OD.

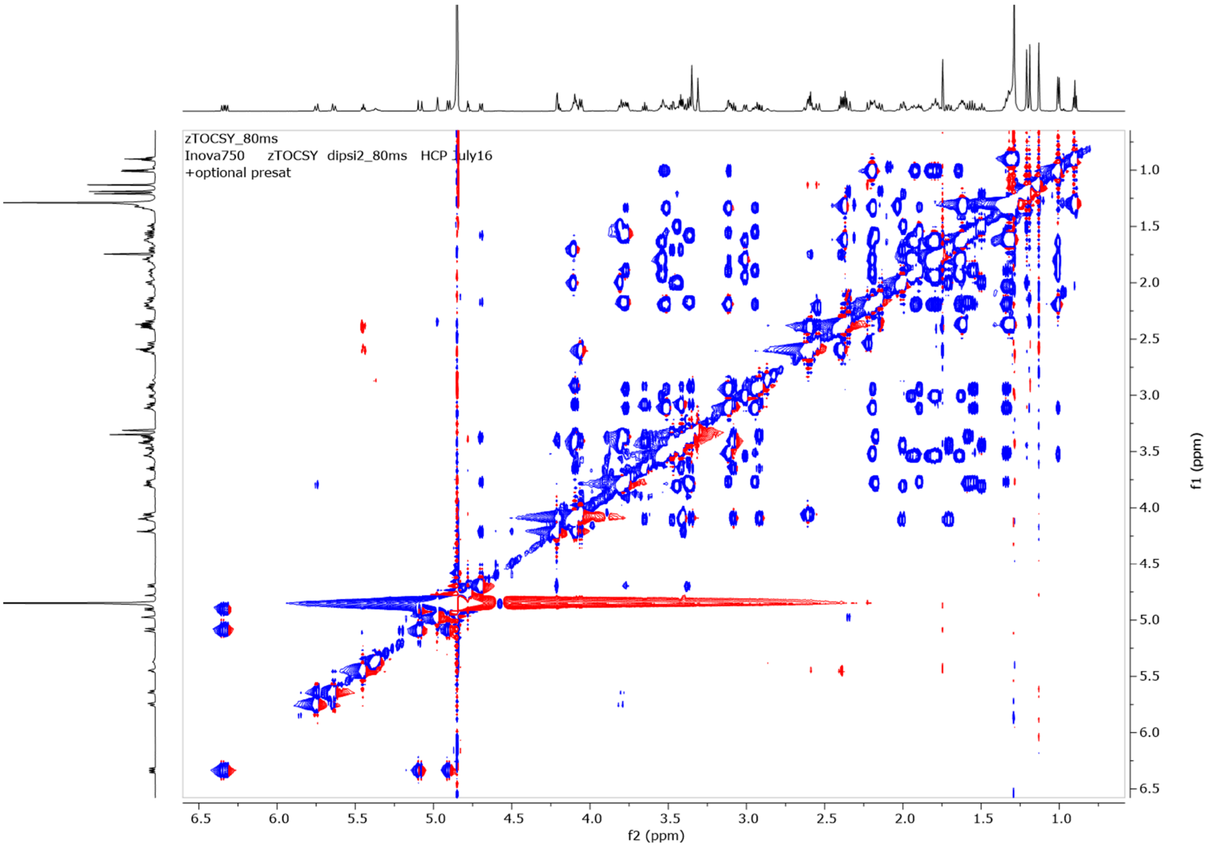

Figure 5. zTOCSY NMR spectrum of **1** at 750 MHz in CD_3OD.

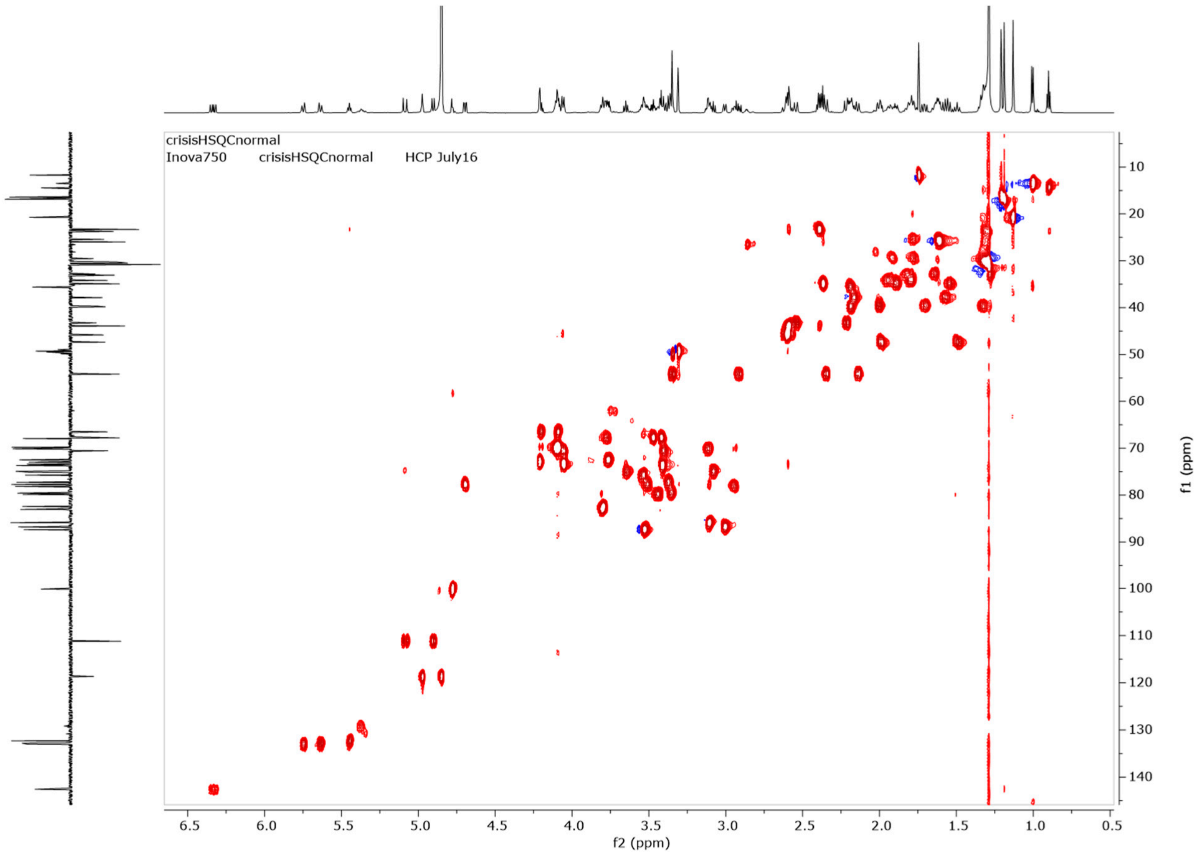

Figure 6. CRISIS-HSQC NMR spectrum of **1** at 750 MHz in CD_3OD.

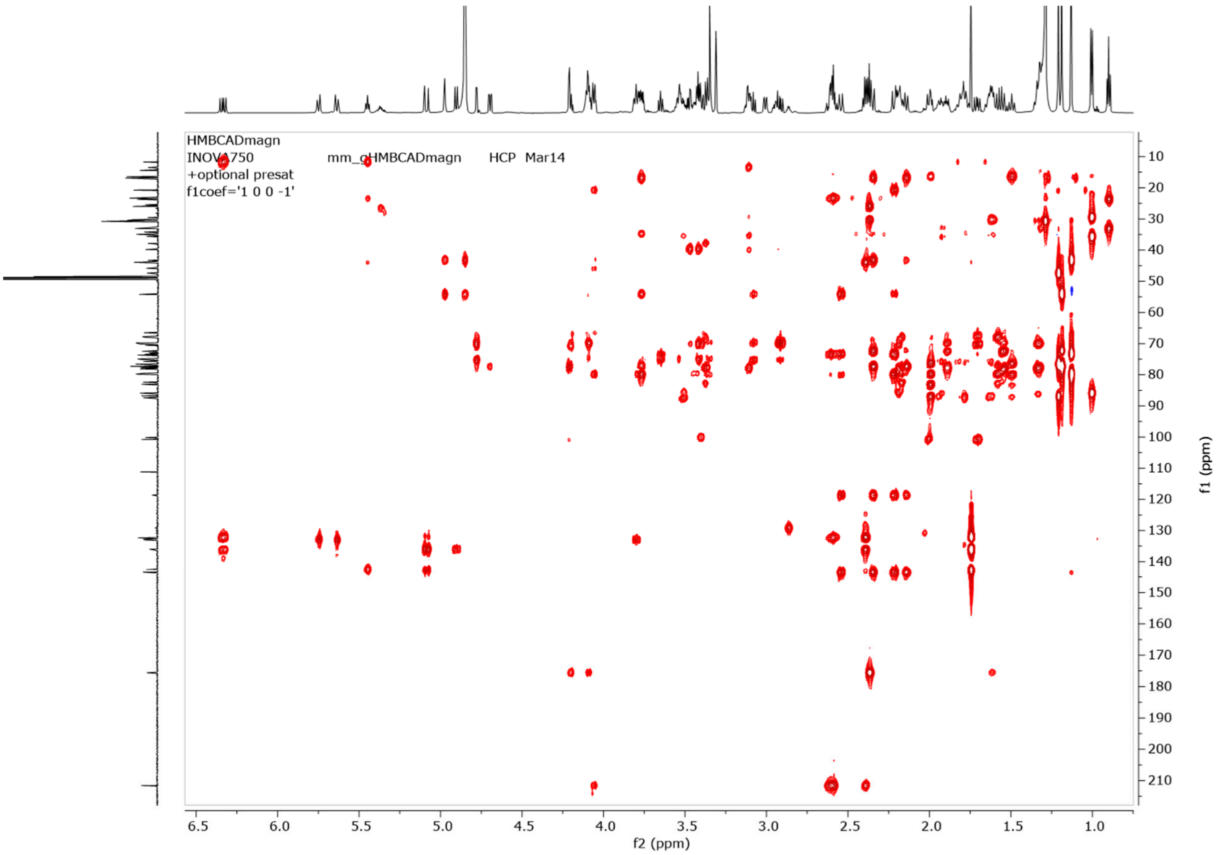

Figure 7. HMBC spectrum of 1 at 750 MHz in CD_3OD.

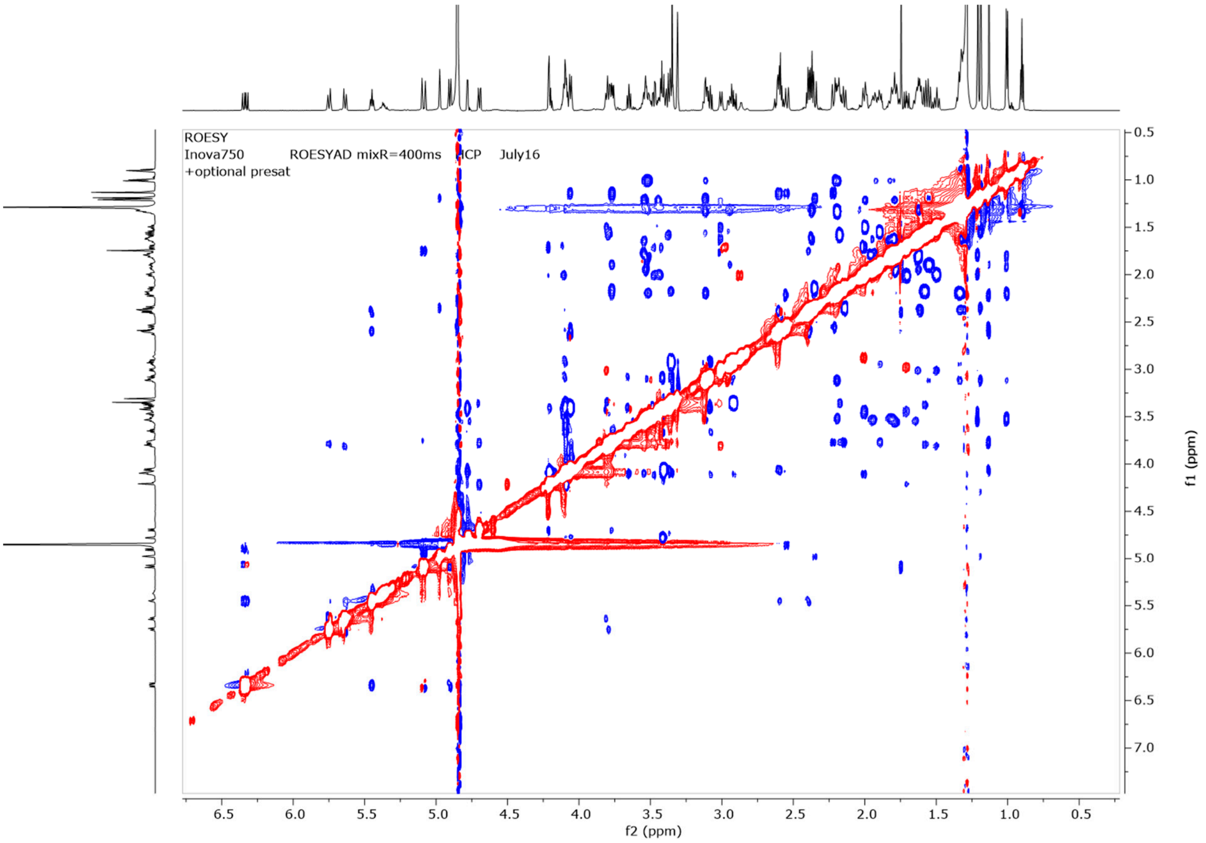

Figure 8. ROESY NMR spectrum of 1 at 750 MHz in CD_3OD.
